# Development of a Sensitive Enzyme Immunoassay Using Phage-Displayed Antigen-Binding Fragments for Zearalenone Detection in Cereal Samples

**DOI:** 10.3390/foods14050746

**Published:** 2025-02-22

**Authors:** Ying Chen, Xinmiao Liu, Jiao Li, Xing Liu

**Affiliations:** School of Food Science and Engineering, Hainan University, Haikou 570228, Chinaliuxinmiao97@163.com (X.L.); lijiaolj0121@163.com (J.L.)

**Keywords:** antigen-binding fragment, cereal, immunoassay, phage display, zearalenone

## Abstract

Zearalenone (ZEN), a non-steroidal estrogenic mycotoxin, contaminates animal feed and grain crops, thereby entering the food chain and posing a significant threat to human health. Consequently, there is an urgent need for a sensitive and rapid method for detecting trace levels of ZEN. In this study, we developed a phage-displayed antigen-binding fragment (Fab-phage) and established a Fab-phage-based enzyme-linked immunosorbent assay (Fab-pELISA) for ZEN detection. Under optimal conditions, this method exhibits a half-maximal inhibitory concentration of 0.36 ng/mL, with a linear range from 0.07 to 3.89 ng/mL and a detection limit of 0.03 ng/mL. The method demonstrates high selectivity towards ZEN and good recovery rates of 97.35–122.66% with relative standard deviations not exceeding 3.5%. Furthermore, the detection results obtained using Fab-pELISA on real cereal samples are consistent with those from high-performance liquid chromatography, meeting practical application requirements. Therefore, the Fab-phage serves as a valuable biochemical reagent, and the established Fab-pELISA represents a promising analytical strategy for detecting ZEN and other trace toxic contaminants in cereals.

## 1. Introduction

Zearalenone (ZEN) is a non-steroidal estrogenic mycotoxin, chemically characterized as 6-[10-hydroxy-6-oxo-trans-1-undecenyl]-β-resorcylactone. It is biosynthesized by various *Fusarium* species, including *Fusarium graminearum* and *Fusarium culmorum*, via the polyketide pathway [1,2,3,4,5]. ZEN primarily contaminates crops such as corn and wheat [6,7,8,9] and exhibits reproductive toxicity, hepatotoxicity, genotoxicity, immunotoxicity, and potential carcinogenicity [10,11,12,13], posing significant risks to human and animal health. Due to its high stability, resistance to high temperatures, and ultraviolet radiation [14,15], ZEN cannot be fully degraded through conventional processing methods. Consequently, the European Food Safety Authority (EFSA) has established a tolerable daily intake of 0.25 μg/kg body weight for adults [16,17]. Additionally, the EU has set maximum limits of 100–200 μg/kg for ZEN in unprocessed cereals and 75 μg/kg in processed cereals [18].

Current quantitative detection methods for ZEN include chromatographic techniques such as high-performance liquid chromatography (HPLC) [19], liquid chromatography tandem mass spectrometry [20], and gas chromatography–mass spectrometry [21], which offer high sensitivity and specificity but are limited by complex sample preparation, high cost, and expensive instrumentation, making them unsuitable for rapid on-site analysis. In contrast, immunoassays provide advantages in terms of speed, cost-effectiveness, ease of operation, and high throughput [22,23], making them an ideal alternative for routine screening. Various immunoassay formats have been developed for mycotoxin detection, including enzyme-linked immunosorbent assay (ELISA) [24], immunosensors [25,26], fluorescence polarization immunoassay [27,28], and lateral flow immunoassay [29,30]. It should be noted that immunosensors, whether employing electrochemical techniques or not, can be conceptually categorized into two main groups: labeled and label-free immunosensors [31].

Monoclonal antibodies (mAbs) are widely utilized in the development of immunoassays for target antigens due to their high sensitivity and specificity. However, the production of mAbs is time-consuming, labor-intensive, and expensive. The development of single-chain variable fragments (scFvs) and antigen-binding fragments (Fabs), which exhibit comparable binding affinity and specificity to mAbs, has mitigated these challenges to some extent [32]. Compared with mAbs, scFvs and Fabs offer additional advantages by reducing non-specific binding through the removal of the crystallizable fragment region [33]. Moreover, their smaller molecular sizes (25 kDa for scFvs and 50 kDa for Fabs) facilitate easier and faster expression and synthesis. Fab fragments consist of a heterodimer of VH-CH1 and VL-CL connected by disulfide bonds [34,35]. The presence of the constant region in Fab enhances the stability of interactions within the complementarity-determining regions (CDRs) between VH and VL domains, making it more stable than scFv [36,37]. Phage display technology provides a robust, straightforward, and cost-effective method for producing miniaturized antibodies, rather than intact antibodies [38,39,40,41]. To date, various miniaturized antibodies, including single-domain antibodies, scFvs, and Fabs, have successfully been developed using phage display technology [42,43,44,45,46,47,48]. Overall, phage display technology not only offers cost-effective amplification and ease of manipulation but also expands the options available for immunoassay development.

**Scheme 1:**
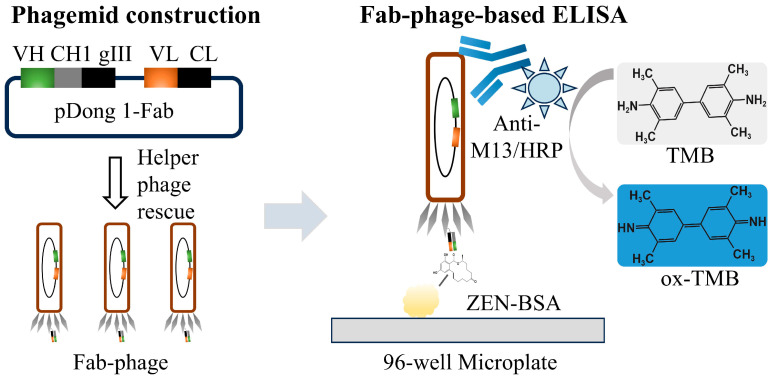
Illustration of Fab-phage-based ELISA.

In this study, we amplified the VL and VH fragments from an anti-ZEN scFv sequence via PCR and subsequently inserted them into the pDong1 vector to construct the pDong1-VL-VH phagemid. This phagemid was then rescued using a helper phage to generate a phage-displayed Fab (Fab-phage). The Fab-phage was utilized to develop a Fab-phage-based ELISA (Fab-pELISA) for detecting ZEN in cereals (Scheme 1). The experimental parameters of the Fab-pELISA were optimized as described in detail in the text. Furthermore, the performance of the Fab-pELISA was comprehensively evaluated based on its detection sensitivity, selectivity, accuracy, and real sample analysis.

## 2. Materials and Methods

### 2.1. Material and Reagents

ZEN, α-Zearalenol, β-Zearalenol, Zearalanone, α-Zearalanol, β-Zearalanol, aflatoxin B_1_ (AFB_1_), fumonisin B_1_ (FB_1_), deoxynivalenol (DON), and ochratoxin A (OTA) standards were procured from Pribolab (Qingdao, China). The ZEN–bovine serum albumin conjugate (ZEN-BSA) and 3,3′,5,5′-tetramethylbenzidine (TMB) were obtained from Sangon Biotech (Shanghai, China). The helper phage M13KO7 was sourced from New England Biolabs (Ipswich, MA, USA). The rabbit Anti-M13 antibody/HRP (Anti-M13/HRP) was acquired from AlpVHH (Chengdu, China). T4 DNA ligase and restriction enzymes (*Sfi* I, *Xho* I, *Sal* I, *Not* I) were purchased from Takara Biomedical Technology (Beijing) Co., Ltd. (Beijing, China). The pDong1 plasmid was kindly provided by Professor Jinhua Dong from the University of Health and Rehabilitation Sciences (Qingdao, China).

### 2.2. Construction of pDong1-VL-VH Phagemid

Using the synthesized pUC-SP-scFv plasmid as a template, the VL and VH fragments were separately amplified by PCR (Tables S1 and S2). Subsequently, the amplified VL fragment and the pDong1 plasmid were co-digested with *Sal* I and *Not* I restriction enzymes, followed by ligation using T4 DNA ligase to generate the pDong1-VL plasmid (Tables S3 and S4). The resulting plasmid was then transformed into *E. coli* DH5α competent cells via heat shock (42 °C for 90 s), and the recombinant clones were verified by colony PCR (Table S5) and DNA sequencing (Sangon Biotech, Shanghai, China). Plasmids from colonies with correct sequences were isolated for further use. For the construction of the pDong1-VL-VH phagemid, the amplified VH fragment and the pDong1-VL plasmid were digested with *Sfi* I and *Xho* I. The larger purified fragments were ligated to produce the pDong1-VL-VH phagemid, following the same procedure used for generating the pDong1-VL plasmid.

### 2.3. Preparation and Characterization of Fab-Phage

The confirmed pDong1-VL-VH phagemid was transformed into *E. coli* TG1 competent cells by heat shock to facilitate the subsequent phage rescue as follows. Briefly, the recombinant bacteria were inoculated in 100 mL of 2 × YT containing 0.1% ampicillin and 2% glucose, followed by cultivation at 37 °C with shaking at 220 rpm to the logarithmic prophase. After adding the helper phage M13KO7 with a multiplicity of infection of 20:1, the culture was left to stand at 37 °C for 15 min, and then incubated at 37 °C with shaking at 220 rpm for 45 min. The culture was subjected to centrifugation (4 °C, 1300× *g*) for 10 min, and the precipitated bacteria were resuspended with 100 mL of 2 × YT containing 0.1% ampicillin and 0.1% kanamycin for cultivation at 30 °C with shaking at 250 rpm for 12 h. After centrifugation (4 °C, 6790× *g*) for 10 min, the supernatant was transferred and mixed with 1/5 volume of 20% PEG/NaCl solution. After incubation at 4 °C for 12 h, the precipitated phage particles in the mixture were collected by centrifugation (4 °C, 10,614× *g*) for 20 min. The pellet was resuspended 1 mL PBS (10 mM, pH 7.4) to obtain the Fab-phage solution, and then the Fab-phage titer was determined using a plate count method. The antigen-binding activity and anti-ZEN reactivity of the Fab-phage were further evaluated by an indirect ELISA and an indirect competitive ELISA, as detailed in the Supplementary Materials.

The confirmed pDong1-VL-VH phagemid was transformed into *E. coli* TG1 competent cells via heat shock to facilitate subsequent phage rescue. Specifically, the recombinant bacteria were inoculated in 100 mL of 2 × YT medium supplemented with 0.1% ampicillin and 2% glucose, and cultured at 37 °C with shaking at 220 rpm until they reached the logarithmic phase. Following this, the helper phage M13KO7 was added at a multiplicity of infection of 20:1, and the culture was incubated statically at 37 °C for 15 min, followed by shaking at 37 °C and 220 rpm for 45 min. The culture was then centrifuged at 4 °C and 1300× *g* for 10 min, and the bacterial pellet was resuspended in 100 mL of 2 × YT medium containing 0.1% ampicillin and 0.1% kanamycin, and further cultivated at 30 °C with shaking at 230× *g* for 12 h. After another centrifugation at 4 °C and 6790× *g* for 10 min, the supernatant was collected and mixed with a 1/5 volume of 20% PEG/NaCl solution. The mixture was incubated at 4 °C for 12 h, after which the precipitated phage particles were collected by centrifugation at 4 °C and 10,614× *g* for 20 min. The resulting pellet was resuspended in 1 mL of PBS (10 mM, pH 7.4) to obtain the Fab-phage solution. The titer of the Fab-phage was determined using a plate count method. Additionally, the antigen-binding activity and anti-ZEN reactivity of the Fab-phage were evaluated using indirect ELISA and indirect competitive ELISA, as detailed in the Supplementary Materials.

### 2.4. Development of the Fab-Phage-Based ELISA for ZEN

Using the Fab-phage as the detection probe, an indirect competitive Fab-phage-based ELISA was developed to detect ZEN as follows. Briefly, a 96-well microplate was coated with a 100 μL/well of ZEN-BSA solution (2 μg/mL in PBS) at 37 °C for 2 h, and then washed three times with PBS containing 0.05% (*v*/*v*) Tween-20 (PBST). The plate was blocked by incubation with 300 μL/well of 3% (*m*/*v*) skim milk powder-PBS solution at 37 °C for 1 h, and then washed three times with PBST. After that, the plate was incubated with 50 μL/well Fab-phage dilution and 50 μL/well of different concentrations of ZEN standard at 37 °C for 1 h. The plate was then washed three times with PBST again, and incubated with 100 μL/well of Anti-M13/HRP (0.2 μg/mL in PBS) at 37 °C for 1 h. After washing another three times with PBST, 100 μL/well of TMB substrate solution was added into the plate for color reaction (37 °C, 10 min), and then 50 μL/well of 2 M H_2_SO_4_ solution was added to terminate the reaction. The absorbance at the wavelength of 450 nm (OD_450_) was recorded by a microplate reader (ST-360, Shanghai Kehua Bio-Engineering Co., Ltd., Shanghai, China). Using a four-parameter logistic equation of Origin2019b (OriginLab, Northampton, MA, USA), the competitive inhibition standard curve was generated, which was fitted by plotting the percentage binding rate as the ordinate against the logarithmic concentration of ZEN standard as the abscissa. The equation binding rate (%) = B/B_0_ × 100% was used to determine the percentage binding rate, where B and B_0_ represent the OD_450_ value in the presence of and absence of ZEN, respectively.

### 2.5. Selectivity of the Fab-pELISA for ZEN

To assess the selectivity of the Fab-pELISA, cross-reactivities (CRs) with ZEN analogs (α-Zearalenol, β-Zearalenol, Zearalanone, α-Zearalanol, β-Zearalanol) and other common mycotoxins (AFB_1_, FB_1_, DON, OTA) were evaluated under optimized experimental conditions. Standard inhibition curves were generated to determine the half-maximal inhibitory concentration (IC_50_). The CR was calculated using the following equation: CR (%) = [(IC_50_ of ZEN)/(IC_50_ of the respective mycotoxin)] × 100%.

### 2.6. Analysis of Cereal Samples and Method Validation

Corn and wheat samples were collected from various local markets in Jiangsu, Shandong, and Hainan Provinces of China. The samples were pre-treated and analyzed as follows: 1 g of the ground cereal sample was weighed and immersed in 5 mL of PBS containing 50% methanol. This mixture was then vigorously shaken for 15 min followed by ultrasonic extraction for an additional 15 min. The resulting solution was centrifuged at 4 °C and 10,614× *g* for 15 min, after which the supernatant was transferred and diluted appropriately with PBS for Fab-pELISA analysis. Negative samples confirmed to be free of ZEN by HPLC (GB5009.209-2016 [49]) were spiked with different concentrations of ZEN (60, 40, and 20 µg/kg), and then extracted and measured following the aforementioned procedure. To further evaluate the effectiveness of Fab-pELISA, real corn and wheat samples were simultaneously pre-treated and analyzed using HPLC for validation. Details of the sample pre-treatment and working conditions for HPLC are provided in the Supplementary Materials.

## 3. Results and Discussion

### 3.1. Construction of pDong1-VL-VH Phagemid and the Preparation and Characterization of Fab-Phage

**Figure 1:**
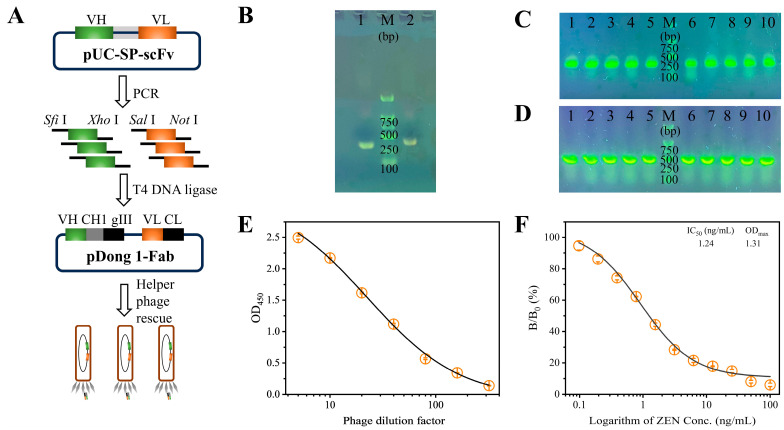
(**A**) Illustration of the construction of pDong1-VL-VH phagemid. Agarose gel electrophoresis analysis of (**B**) amplified VL fragments (lane 1) and VH fragments (lane 2), (**C**) pDong1-VL colony PCR products (lanes 1–10), and (**D**) pDong1-VL-VH colony PCR products (lanes 1–10); M: D2000 DNA marker (Sangon Biotech, Shanghai, China). Analysis of the antigen-binding activity (**E**) and anti-ZEN reactivity (**F**) of the Fab-phage. Error bars represent the standard deviations derived from three independent replicates.

The construction of the pDong1-VL-VH phagemid was carried out in a stepwise manner, as illustrated in Figure 1A. The VL and VH fragments were amplified via PCR, and the resulting products were analyzed using 1% agarose gel electrophoresis. As shown in Figure 1B, two distinct bands of approximately 320 bp (VL) and 360 bp (VH) were observed, confirming the successful amplification of the VL and VH fragments. Subsequently, the purified VL and VH fragments were sequentially inserted into the pDong1 plasmid to generate the intermediate pDong1-VL plasmid and the final pDong1-VL-VH phagemid. Both constructs were introduced into *E. coli* DH5α competent cells by heat shock transformation, followed by plating for antibiotic resistance screening. As demonstrated in Figure 1C,D, randomly selected colonies were verified as positive by colony PCR, providing preliminary evidence of successful construction. Furthermore, these findings were validated through DNA sequencing, thereby confirming the successful assembly of the pDong1-VL-VH phagemid.

The pDong1-VL-VH phagemid was transformed into *E. coli* TG1 competent cells and subsequently rescued by the M13KO7 helper phage to generate the Fab-phage library. The resulting Fab-phage titer was determined to be 6 × 10^11^ cfu/mL. The antigen-binding activity of the Fab-phage was evaluated using an indirect ELISA. As illustrated in Figure 1E, the OD_450_ value decreased from 2.50 to 0.14 as the phage dilution factor increased from 5 to 320, indicating robust antigen-binding activity. Furthermore, the anti-ZEN reactivity of the Fab-phage was assessed via an indirect competitive ELISA. As shown in Figure 1F, the percentage binding rate decreased with increasing concentrations of ZEN standard, demonstrating that the coated ZEN-BSA competes effectively with free ZEN for binding to the Fab-phage. A typical sigmoidal competitive inhibition curve was generated, with an IC_50_ value of 1.24 ng/mL, confirming the high sensitivity of the Fab-phage towards ZEN. Therefore, the prepared Fab-phage is suitable for developing a sensitive immunoassay for ZEN detection.

### 3.2. Optimization of Fab-pELISA for ZEN

**Figure 2:**
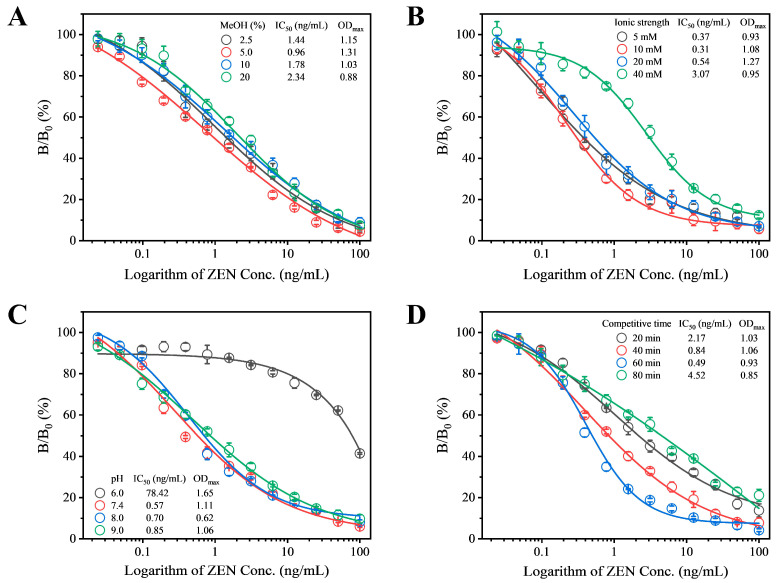
Evaluation of (**A**) methanol concentration, (**B**) ionic strength, (**C**) pH, and (**D**) competitive reaction time on the performance of Fab-pELISA. Error bars represent the standard deviations derived from three independent replicates.

To enhance the detection sensitivity of Fab-pELISA, a comprehensive optimization of experimental parameters was conducted. These parameters included methanol concentration, ionic strength, pH, and competitive time, with IC_50_ and OD_max_ (OD_450_ in the absence of ZEN) serving as evaluation criteria. Prior to this optimization, checkerboard titration (Table S6) determined the concentrations of ZEN-BSA and Fab-phage to be 1.0 μg/mL and 1.5 × 10^10^ cfu/mL, respectively. ZEN exhibits strong polarity and excellent fat solubility. Therefore, a specific proportion of methanol is typically added during the preparation of ZEN standards and sample extraction. The concentration of methanol may also influence the interaction between antigen and antibody; hence, we evaluated the methanol concentration. When the methanol concentration was 5%, the IC_50_ value reached its lowest point at 0.96 ng/mL, thus 5% methanol concentration was chosen for subsequent experiments (Red circle, Figure 2A). Additionally, variations in ionic strength, pH, and competition time within the competitive system can influence the specific binding between antigen and antibody, thereby affecting detection sensitivity. Therefore, these parameters were optimized step by step. Among the tested ionic strengths of 2.5 mM, 5.0 mM, 10 mM, and 20 mM, the lowest IC_50_ value of 0.31 ng/mL was observed at 10 mM (Red circle, Figure 2B), leading to the selection of 10 mM as the optimal ionic strength for the reaction system. Under different pH conditions ranging from 6.0 to 9.0, the lowest IC_50_ value was observed at pH 7.4 (Red circle, Figure 2C), indicating that pH 7.4 was the most suitable reaction condition. The IC_50_ value continuously decreased from 20 min to 60 min but increased when the time extended beyond 60 min to 80 min, hence 60 min was selected as the optimal competitive reaction time (Blue circle, Figure 2D). After optimization, the final parameters for the Fab-pELISA were determined as follows: ZEN-BSA concentration of 1 μg/mL, Fab-phage concentration of 1.5 × 10^10^ cfu/mL, methanol concentration of 5%, ionic strength of 10 mM, pH of 7.4, and competitive reaction time of 60 min.

### 3.3. Analytical Performance of the Fab-pELISA for ZEN

**Figure 3:**
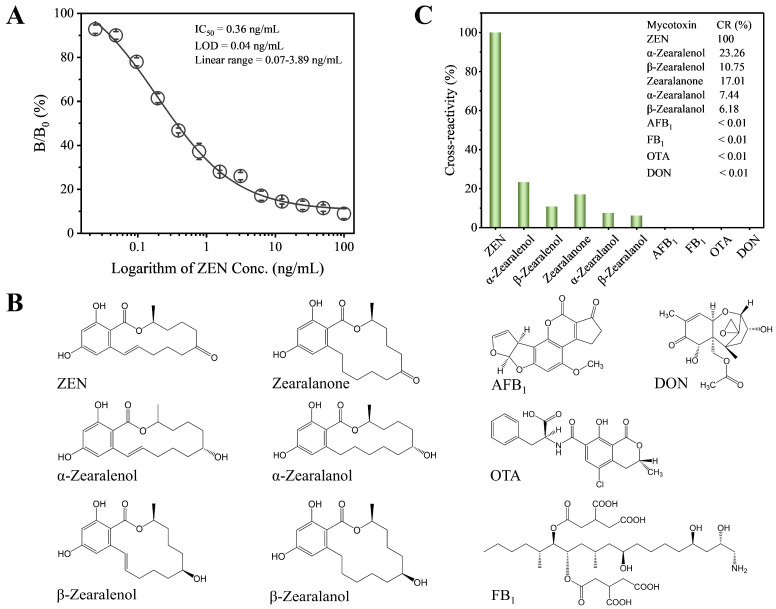
(**A**) The standard competitive inhibition curve of Fab-pELISA. (**B**) Chemical structures of ZEN, α-zearalenol, β-zearalenol, zearalanone, α-zearalanol, β-zearalanol, AFB_1_, FB_1_, OTA, and DON. (**C**) Histogram analysis of cross-reactivity rates for ZEN, α-zearalenol, β-zearalenol, zearalanone, α-zearalanol, β-zearalanol, AFB_1_, FB_1_, OTA, and DON. Error bars represent standard deviations derived from three independent replicates.

Under the optimized conditions, we established the standard competitive inhibition curve of Fab-pELISA (Figure 3A), which exhibits an IC_50_ of 0.36 ng/mL, a linear range (IC_20_-IC_80_) of 0.07–3.89 ng/mL, and a limit of detection (IC_10_) of 0.03 ng/mL. Compared to most reported immunoassays for ZEN [23,28,46,47,48,50,51,52,53,54,55,56,57,58], including colorimetric, fluorescent, bioluminescent, and chemiluminescent formats (Table 1), the Fab-pELISA demonstrates either higher or comparable detection sensitivity. Moreover, the Fab-pELISA eliminates the need for mAbs and the complex synthesis of nanomaterials. However, incorporating various nanomaterials into immunoassays [48,51,52,53,57] can offer strategies to further enhance the detection sensitivity of Fab-pELISA. Several immunoassays for ZEN utilize mimotope peptides and anti-idiotypic nanobodies as non-toxic substitutes for ZEN [51,53,55,56,57,58]. From a sustainable technology perspective, both mimotope peptides and anti-idiotypic nanobodies can be produced through prokaryotic expression and one-step purification, making the Fab-pELISA more stable, cost-effective, and environmentally friendly when replacing ZEN-BSA with these alternatives.

### 3.4. Selectivity of the Fab-pELISA

The selectivity of the Fab-pELISA was assessed by evaluating CRs with ZEN analogs and other mycotoxins (Figure 3B). The assay demonstrated high specificity for ZEN, with relatively low CRs of 23.26% for α-zearalenol, 10.75% for β-zearalenol, 17.01% for zearalanone, 7.44% for α-zearalanol, and 6.18% for β-zearalanol. In contrast, negligible CRs (<0.01%) were observed for AFB_1_, FB_1_, OTA, and DON (Figure 3C). These results confirm the high selectivity of the Fab-pELISA for ZEN. To further validate the robustness of the assay, it would be beneficial to test a wider range of interfering substances, including additional mycotoxins and other food contaminants, thereby providing a more comprehensive evaluation of its selectivity.

### 3.5. Analysis of Cereal Samples and Validation

**Figure 4:**
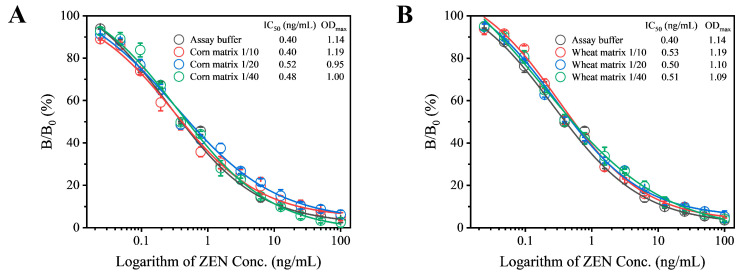
Evaluation of matrix effects from corn (**A**) and wheat (**B**) on the performance of Fab-pELISA. Error bars represent the standard deviations derived from three independent replicates.

In this study, corn and wheat were selected as sample substrates to evaluate the matrix effect as described in the Supplementary Materials. The extracts from these negative samples were diluted at ratios of 10, 20, and 40 times, with the final diluted extract containing a methanol concentration of 5%. A series of ZEN standards were prepared using these extracts. Under optimized experimental conditions, the standard competitive inhibition curves for sample analysis were established. When corn and wheat samples were diluted by 10 and 20 times, respectively, the resulting standard curves showed good agreement with those obtained in the absence of matrix effects (Figure 4). Therefore, for subsequent analyses, corn samples were diluted 10 times and wheat samples 20 times. When applying this assay to complex sample matrices beyond cereals, such as other food or feed types, potential challenges should be further elaborated, which may require additional optimization.

The accuracy of the Fab-pELISA, encompassing both trueness and precision, was evaluated through intra-assay and inter-assay spike-and-recovery experiments conducted on negative corn and wheat samples. Three spiking levels of ZEN (10, 20, and 40 μg/kg) were used for these evaluations. For spiked corn samples, the intra-assay recovery rates ranged from 103.28% to 107.33%, with relative standard deviations (RSDs) between 1.89% and 2.42%. The inter-assay recovery rates ranged from 100.32% to 124.64%, with RSDs between 1.33% and 2.63% (Table 2). For spiked wheat samples, the intra-assay recovery rates were between 98.60% and 115.60%, with RSDs ranging from 1.27% to 3.17%. The inter-assay recovery rates were between 97.35% and 122.66%, with RSDs ranging from 1.12% to 3.28% (Table 2). These results collectively demonstrate the high accuracy of the Fab-pELISA.

To further validate the effectiveness of the method in analyzing real samples, the ZEN content in ten corn and six wheat samples was determined using Fab-pELISA and concurrently verified by HPLC. The measured ZEN concentrations ranged from 4.49 to 738.96 μg/kg for Fab-pELISA and from 5.40 to 745.54 μg/kg for HPLC (Table 3). As illustrated in Figure S1, the detection results obtained by Fab-pELISA and HPLC exhibited a strong correlation, as represented by the linear regression equation y = 0.93471 + 0.91415x (R^2^ = 0.99). These findings confirm that the developed Fab-pELISA is a reliable method for the quantitative determination of ZEN in cereal samples. Additionally, a detailed comparison of the overall cost of Fab-pELISA versus HPLC was described in the Supplementary Materials and summarized in Table S7, further verifying the application potential of Fab-pELISA.

## 4. Conclusions

Thus, we developed a Fab-phage-based enzyme immunoassay (Fab-pELISA) for detecting ZEN in cereal samples using a constructed Fab-phage. The optimized Fab-pELISA exhibited high sensitivity and selectivity for ZEN detection in cereal samples, with an IC_50_ of 0.36 ng/mL and a limit of detection of 0.03 ng/mL. Importantly, this study underscores the potential application of Fab-phage technology in mycotoxin immunoassays. By utilizing Fab-phages, the need for producing mAbs via hybridoma technology can be circumvented, thereby significantly reducing both time and cost. Given that Fab-phages retain robust antibody activity and phage particles serve as natural carriers, it is feasible to integrate various nanomaterials with Fab-phages, leading to the development of advanced immunosensors with enhanced analytical performance. Furthermore, the Fab-pELISA can be scalable with automation, such as robotic plate handlers. However, due to inconsistent display efficiency across different batches of Fab-phage amplification, its current market application is limited. In the future, optimizing amplification protocols and improving phage display efficiency could facilitate the development of a commercial kit for broader market application.

## Figures and Tables

**Table 1:** Comparison of the Fab-pELISA with other reported immunoassays for ZEN.

Methods	Used Materials	IC_50_ (ng/mL)	LOD (ng/mL)	Refs.
ELISA	scFv	90	20	[23]
ELISA	mAb, gold nanorod	0.85	0.22	[50]
ELISA	mAb	8.69	0.76	[51]
LFIA	mAb, polydopamine-coated gold nanoparticles	-	0.0074	[52]
MB-ELISA	mAb, magnetic bead	1.78	0.13	[53]
Fluorescent ELISA	mAb, phage mimotope, quantum dot	0.301	0.023	[54]
Fluorescent ELISA	mAb, quantum dot	0.075	0.0041	[55]
Fluorescent ELISA	mAb, mimotope peptide, upconversion nanoparticles	0.16	0.02	[56]
Fluorescent LFIA	mAb, quantum dot	2.792	0.273	[57]
Fluorescent LFIA	mAb, quantum dot	-	0.58	[29]
Fluorescent LFIA	mAb, anti-idiotypic nanobody, Eu/Tb(III) nanosphere	0.52	0.07	[58]
Immuno-PCR	mAb, phage-displayed anti-idiotypic nanobody	0.257	0.08	[59]
Chemiluminescent ELISA	mAb, phage mimotope, magnetic bead	0.0314	0.0043	[60]
Bioluminescent immunosensor	mAb, mimotope peptide-*Gaussia* luciferase fusion protein, protein G–coupled magnetic beads	11.0	4.2	[61]
pELISA	Fab-phage	0.36	0.03	This work

**Table 2:** Recoveries of ZEN from spiked samples determined by the Fab-pELISA.

Sample	Spiked Level (µg/kg)	Intra-Assay (*n* = 3) ^a^			Inter-Assay (*n* = 3) ^b^		
		Mean ± SD (µg/kg)	Recovery (%)	RSD (%)	Mean ± SD (µg/kg)	Recovery (%)	RSD (%)
Corn	10	10.73 ± 0.20	107.33	1.89	12.06 ± 0.16	120.64	1.33
	20	20.65 ± 0.43	103.28	2.11	20.06 ± 0.39	100.32	1.99
	40	41.77 ± 1.01	104.43	2.42	42.00 ± 1.10	105.02	2.63
Wheat	10	11.56 ± 0.15	115.60	1.29	12.26 ± 0.13	122.66	1.12
	20	22.38 ± 0.29	111.90	1.27	19.47 ± 0.36	97.35	3.28
	40	39.44 ± 1.25	98.60	3.17	44.34 ± 0.89	110.86	2.02

^a^ Each assay was conducted in triplicate on the same day. ^b^ Each assay was conducted in triplicate on three separate days.

**Table 3:** Analysis of ZEN contents in cereal samples by Fab-pELISA and HPLC.

Sample	Fab-pELISA (*n* = 3)		HPLC (*n* = 3) [62]	
	Mean ± SD (µg/kg)	RSD (%)	Mean ± SD (µg/kg)	RSD (%)
Wheat-1	7.60 ± 0.11	1.45	8.21 ± 0.52	6.33
Wheat-2	Not detected	-	Not detected	-
Wheat-3	4.59 ± 0.01	0.22	5.40 ± 0.08	1.48
Wheat-4	10.66 ± 0.23	2.16	9.53 ± 0.38	3.99
Wheat-5	141.95 ± 1.70	1.20	129.11 ± 1.04	0.81
Wheat-6	142.42 ± 1.34	0.94	149.01 ± 3.14	2.11
Wheat-7	12.31 ± 0.25	2.03	11.83 ± 0.17	1.44
Wheat-8	211.02 ± 5.38	2.55	199.74 ± 2.38	1.19
Wheat-9	168.55 ± 4.70	2.79	164.21 ± 2.35	1.43
Wheat-10	180.62 ± 2.21	1.22	186.36 ± 4.90	2.63
Corn-1	Not detected	-	Not detected	-
Corn-2	33.62 ± 0.67	1.99	27.33 ± 0.36	1.32
Corn-3	6.56 ± 0.13	1.98	7.22 ± 0.42	5.82
Corn-4	32.39 ± 0.99	3.06	33.18 ± 0.93	2.80
Corn-5	738.96 ± 6.88	0.93	745.54 ± 20.31	2.72
Corn-6	83.12 ± 1.72	2.07	95.95 ± 6.43	6.70

## Data Availability

The original contributions presented in the study are included in the article/Supplementary Materials; further inquiries can be directed to the corresponding authors.

## Supplementary Materials

The following supporting information can be downloaded at https://www.mdpi.com/article/10.3390/foods14050746/s1: Characterization of the Fab-phage; Evaluation of the matrix effects of cereal samples; Analysis of ZEN by HPLC [49]; Table S1: primer sequences for the amplification of VH and VL fragments; Table S2: PCR system and procedure for VH and VL fragments; Table S3: double-enzyme digestion system and procedure for VL fragment and pDong1 plasmid; Table S4: ligation system and procedure for pDong1-VL; Table S5: colony PCR system and procedure for pDong1-VL; Table S6: optimization of concentrations of ZEN-BSA and Fab-phage by checkerboard titration; Table S7: Summary of cost comparison between Fab-pELISA and HPLC; Figure S1: linear regression analysis of the correlation between the detection results of Fab-pELISA and HPLC.
