# Supplementary material for: Development of a Sensitive Enzyme Immunoassay Using Phage-Displayed Antigen-Binding Fragments for Zearalenone Detection in Cereal Samples
Source: Foods. 2025 Feb 22;14(5):746. doi: 10.3390/foods14050746 (PMC11898766; doi:10.3390/foods14050746)
Supplement: Supplementary file 1 [file foods-14-00746-s001.zip › foods-3470768-supplementary.pdf]

Supplemental Material for

Development of a Sensitive Enzyme Immunoassay Using Phage-Displayed Antigen-Binding Fragments for Zearalenone Detection in Cereal Samples

Ying Chen †, Xinmiao Liu †, Jiao Li and Xing Liu *

School of Food Science and Engineering, Hainan University, Haikou 570228, China; yingchen@hainanu.edu.cn (Y.C.); liuxinmiao97@163.com (X.L.); lijiaolj0121@163.com (J.L.)

* Correspondence: xliu@hainanu.edu.cn

† These authors contributed equally to this work.

Contents

Characterization of the Fab-phage--S-3

Evaluation of the Matrix Effects of Cereal Samples-----------------------------------S-4

Analysis of ZEN by HPLC---S-5

Table S1--S-6

Table S2--S-7

Table S3--S-8

Table S4--S-9

Table S5--S-10

Table S6--S-11

Figure S1--S-12

Analysis of detailed cost comparison between Fab-pELISA and HPLC-------------S-13

Table S7--S-16

Characterization of the Fab-phage

To evaluate the antigen-binding activity and anti-ZEN reactivity of the Fab-phage, an indirect ELISA was conducted as follows. A 96-well microplate pre-coated with 0.2 μg ZEN-BSA per well was incubated with a series of Fab-phage dilutions at 37 °C for 1 h. Following three washes with PBS containing 0.05% (v/v) Tween-20 (PBST), the plate was incubated with Anti-M13/HRP (300 μL/well, 0.2 μg/mL in PBS) at 37 °C for 1 h. After another three washes with PBST, the plate was incubated with TMB substrate solution (100 μL/well) for color development at 37 °C for 10 min. The reaction was stopped by adding 100 μL/well of 2 M H_2SO_4, and the absorbance, at 450 nm (OD_{450}), was measured using a microplate reader (ST-360, Shanghai Kehua Bio-Engineering Co., Ltd., Shanghai, China). Additionally, the Fab-phage reactivity was assessed using an indirect competitive ELISA. This procedure followed the same steps as the indirect ELISA described above, except that the Fab-phage dilutions were replaced with 50 μL/well of phage dilution and 50 μL/well of varying concentrations of ZEN standard.

Evaluation of the matrix effects of cereal samples

The sample matrix effect can interfere with antigen–antibody interactions, thereby compromising the detection accuracy of immunoassays. In this study, corn and wheat samples used for evaluating the matrix effect were sourced from local markets in Haikou City, China, and confirmed to be ZEN-negative via HPLC (GB5009.209-2016). Sample pretreatment and matrix effect evaluations were performed as follows: 1 g of ground cereal sample was weighed and suspended in 5 mL of PBS containing 50% methanol. The mixture was then vigorously shaken for 15 min followed by ultrasonic extraction for an additional 15 min. Subsequently, the mixture was centrifuged at 4 °C and 10,614× *g* for 15 min, after which the supernatant was filtered through a 0.22 μm filter membrane. The filtered extracts were serially diluted and utilized to prepare a series of ZEN standard concentrations for Fab-pELISA analysis. The inhibition standard curves were constructed as detailed in Section 2.4.

Analysis of ZEN by HPLC

The sample was pretreated and tested according to a modified version of the previously described method [1]. Specifically, 5.0 g of ground sample (accurate to 0.1 g) was weighed into a 50 mL centrifuge tube containing 1 g NaCl and 10 mL of 90% acetonitrile-water solution (v/v). The mixture was extracted by high-speed homogenization for 2 min using a homogenizer (HFJ-10, HengAo Technology, Tianjin, China). Following centrifugation at 2653× *g* for 10 min, the supernatant was filtered through quantitative filter paper with a pore size of 1.6 μm. Subsequently, 5.0 mL of the filtrate was diluted with 20 mL of water. The diluted sample solution was then passed through a GF/A Whatman glass fiber filter (GE Healthcare, Maidstone, UK), and the filtrate was stored at 4 °C until HPLC analysis. HPLC analysis was conducted using a Waters 2695 separation system equipped with a Waters 2469 fluorescence detector (Waters, Milford, MA, USA). The detection conditions were as follows: 1) The mobile phase consisted of acetonitrile/methanol/water in a volume ratio of 46:8:46; 2) The excitation and emission wavelengths for ZEN detection were set at 274 nm and 440 nm, respectively; 3) Chromatographic separation of ZEN was achieved on a Waters XBridge C18 column (4.6 × 250 mm, 5 μm) at a flow rate of 1.0 mL/min, with the column temperature maintained at room temperature.

Table S1. Primer sequences for the amplification of VH and VL fragments.

Name	Sequence (5'-3')
VH-F-Sfi I	TATTACTCGCGGCCCAGCCGGCCATGGCCCAGGTGAAACTGCAG
VH-R-Xho I	CCGCTCGAGGAGGAGACGGTGACCGTGGTCCCTTGGCCCC
VL-F-Sal I	ACGCGTCGACGGACATTGAGCTCACCCAGTCTCCA
VL-R-Not I	GAGTCATTCTGCGGCCGCCCGTTTGATTTCCAGCTTGGTCCC

Table S2. PCR system and procedure for VH and VL fragments.

Components	Volume (μL)
Primer: VL-F-Sal I or VH-F-Sfi I	2.0 (10 μM)
Primer: VL-R-Not I or VH-R-Xho I	2.0 (10 μM)
2 × Phanta Flash Master Mix (Dye Plus)	25
pUC-SP-scFv	1.0
ddH_2O	20

Reaction conditions: Predenaturation at 98 °C for 30 s; 30 cycles of denaturation at 98 °C for 10 s, annealing at 65 °C for 5 s, and extension at 72 °C for 5 s; thorough extension at 72 °C for 1 min.

Table S3. Double-enzyme digestion system and procedure for VL fragment and pDong1 plasmid.

Components	Volume (μL)
10 × H buffer	5.0
0.1% BSA	5.0
Not I	2.5
Sal I	2.5
pDong/VL	X (≤ 2.5 μg)
ddH_2O	Up to100

Reaction conditions: 37 °C, 2.5 h.

Table S4. Ligation system and procedure for pDong1-VL.

Components	Volume (μL)
Solution I	X + Y (5-10 μL)
VL (*Not* I/*Sal* I)	X (0.03-0.3 pmol)
pDong1 (*Not* I/*Sal* I)	Y (0.03 pmol)

Reaction conditions: 16 ℃, 30 min.

Table S5. Colony PCR system and procedure for pDong1-VL.

Components	Volume (μL)
Primer: VL-Sal I	1.0 (10 μM)
Primer: VL-Not I	1.0 (10 μM)
2 × Rapid Taq Master Mix	12.5
Bacterial supernatant	1.0
ddH_2O	9.5

Reaction conditions: Predenaturation at 95 °C for 3 min; 30 cycles of denaturation at 95 °C for 15 s, annealing at 60 °C for 15 s, and extension at 72 °C for 7 s; thorough extension at 72 °C for 5 min.

Table S6. Optimization of concentrations of ZEN-BSA and Fab-phage by checkerboard titration.

Fab-phage	ZEN-BSA Conc. (μg/mL)			
Conc. (cfu/mL)	4.0	2.0	1.0	0.5
3×10^{10}	3.35	3.06	1.68	1.14
1.5×10^{10}	3.21	2.91	**1.44**	1.08
7.5×10^{9}	3.18	2.24	0.82	0.67
3.75×10^{9}	2.89	1.50	0.59	0.46
1.875×10^{9}	1.95	0.87	0.36	0.34
9.375×10^{8}	1.01	0.54	0.34	0.34
4.6875×10^{8}	0.48	0.29	0.27	0.22
Blank	0.18	0.21	0.16	0.17

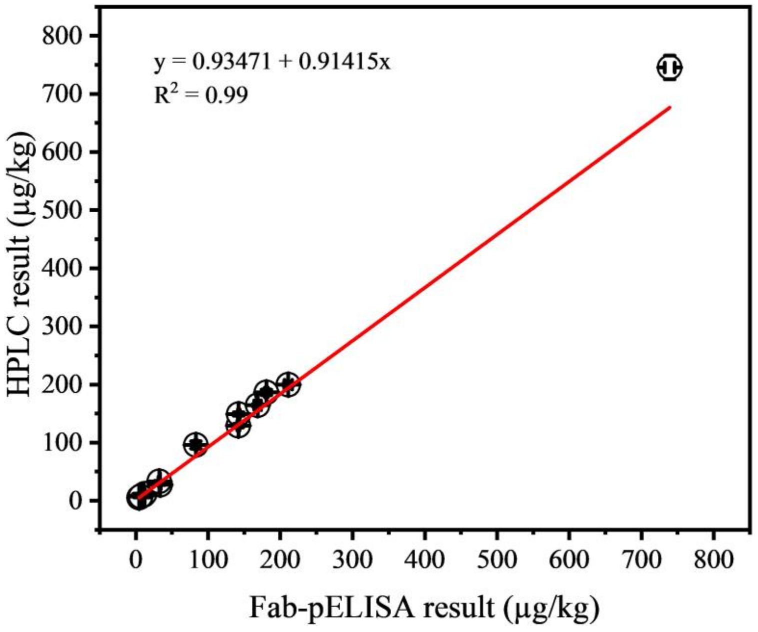

Figure S1. Linear regression analysis of the correlation between the detection results of Fab-pELISA and HPLC. Error bars represent the standard deviations derived from three independent replicates.

Analysis of detailed cost comparison between Fab-pELISA and HPLC

The cost comparison involves initial setup costs, operational costs, personnel and expertise, and throughput and scalability.

1. Initial setup costs

1.1 Fab-pELISA:

a) Requires phage-displayed antibody libraries, which involve upfront costs for library construction, immunization, and screening.

b) Equipment includes microplate readers, incubators, and basic ELISA consumables (e.g., 96-well plates, pipettes). These are generally lower-cost compared to HPLC systems.

HPLC:

a) High initial investment in instrumentation (chromatography systems, detectors, columns) and specialized software.

b) Columns and solvents (e.g., acetonitrile, buffers) add recurring costs. For example, columns degrade over time and require replacement, which is costly.

2. Operational costs

2.1 Fab-pELISA:

a) Lower per-test costs due to batch processing (e.g., 96 samples per plate) and reusable phage libraries.

b) Consumables include antibodies, enzyme conjugates (e.g., HRP), and substrates (e.g., TMB). For instance, an ELISA Kit commonly lists ~500–1000 USD for 96 tests.

c) Minimal solvent use reduces waste disposal costs.

2.2 HPLC:

a) Higher per-sample costs due to solvents, column maintenance, and energy consumption. For example: A single HPLC run can cost 10–50 USD depending on solvent usage and column lifespan.

b) Method development (e.g., optimizing gradients for new analytes) adds labor and resource expenses.

3. Personnel and expertise

3.1 Fab-pELISA:

a) Requires training in immunoassay techniques (e.g., pipetting, plate washing) but is relatively standardized.

b) Minimal specialized expertise compared to HPLC.

3.2 HPLC:

a) Demands skilled technicians for method optimization, troubleshooting, and data analysis.

b) Training costs are higher, and errors (e.g., column contamination) can lead to costly downtime.

4. Throughput and scalability

4.1 Fab-pELISA:

a) High throughput: 96 samples processed in 2–4 hours, ideal for large-scale screening.

b) Scalable with automation (e.g., robotic plate handlers).

4.2 HPLC:

a) Lower throughput: Typically 10–20 samples per day due to longer run times (30–60 minutes per sample).

b) Limited scalability without additional instruments.

Table S7. Summary of cost comparison between Fab-pELISA and HPLC.

**Factor**	**Fab-pELISA**	**HPLC**
Initial setup	5,000–20,000 USD	50,000–200,000+ USD
Per-test cost	5–20 USD	10–50+ USD
Throughput	High (96 samples/plate)	Low–Moderate
Maintenance	Low (reagents, plate readers)	High (columns, solvents, instrumentation)
Expertise required	Moderate	High

References

[1] Zhang, B.; Li, H.; Li, Y.; Fu, X.; Du, D. A sensitive chemiluminescence immunoassay based on immunomagnetic beads for quantitative detection of zearalenone. *Eur. Food Res. Technol.* **2021,** *247*(9), 2171-2181.
